# Secretion of MCP-1 and other paracrine factors in a novel tumor-bone coculture model

**DOI:** 10.1186/1471-2407-9-45

**Published:** 2009-02-03

**Authors:** Katherine R Schiller, Marion R Zillhardt, Jeremy Alley, Dori L Borjesson, Alvin J Beitz, Laura J Mauro

**Affiliations:** 1 Department of Animal Science-Physiology, University of Minnesota, St. Paul, MN 55108-6009, USA; 2 Department of Veterinary & Biomedical Sciences, University of Minnesota, St. Paul, MN 55108-6009, USA; 3 Department of Pathology, Microbiology & Immunology, University of California-Davis, Davis, CA 95616-8500, USA

## Abstract

**Background:**

The bone-tumor microenvironment encompasses unique interactions between the normal cells of the bone and marrow cavity and the malignant cells from a primary or metastasized cancer. A multitude of paracrine factors within this microenvironment such as the growth factor, TGF-β, and the chemokine, MCP-1, are secreted by many of these cell types. These factors can act in concert to modulate normal and malignant cell proliferation, malignant cell migration and invasion and, often, mediate bone cancer pain. Although many valuable *in vitro *and *in vivo *models exist, identifying the relevant paracrine factors and deciphering their interactions is still a challenge. The aim of our study is to test an ex vivo coculture model that will allow monitoring of the expression, release and regulation of paracrine factors during interactions of an intact femur explant and tumor cells.

**Methods:**

Intact or marrow-depleted neonatal mouse femurs and select murine and human sarcoma or carcinoma cell lines were incubated singly or in coculture in specialized well plates. Viability of the bone and cells was determined by immunohistochemical stains, microscopy and marrow cytopreps. Secretion and mRNA expression of paracrine factors was quantitated by ELISA and real-time RT-PCR.

**Results:**

Compartments of the bone were optimally viable for up to 48 h in culture and tumor cells for up to 4 days. Bone was the major contributor of TGF-β and MMP2 whereas both bone and sarcoma cells secreted the chemokine MCP-1 in cocultures. Synergistic interaction between the femur and sarcoma resulted in enhanced MCP-1 secretion and expression in cocultures and was dependent on the presence of the hematopoietic component of the bone as well as other bone cells. In contrast, coculturing with breast carcinoma cells resulted in reduction of TGF-β and MCP-1 secretion from the bone.

**Conclusion:**

These studies illustrate the feasibility of this model to examine paracrine interactions between intact bone and tumor cells. Further study of unique regulation of MCP-1 secretion and signaling between these cell types in different types of cancer will be possible using this simulated microenvironment.

## Background

Cancer is a disease whose outcome is determined by the malignant tumor cells themselves as well as by the microenvironment in which they reside. The initial cellular oncogenic transformation is due to the acquisition or inheritance of genetic mutations which endows these cells with a malignant phenotype. The subsequent successful progression of a tumor also requires favorable tumor-host interactions. Within the tumor microenvironment, it is the non-malignant cells, often termed the 'stroma', which are active and essential components that are recruited and exploited by malignant cells to ensure tumor survival and growth [1]. This is also true during metastasis when invasive malignant cells must colonize a 'foreign' microenvironment and establish a secondary metastatic tumor [2]. It is the paracrine factors of this reactive stroma which direct the communication between the malignant and non-malignant cells and therefore are important regulators of this microenvironment.

The bone is a unique and complex microenvironment that serves as a primary site for sarcomas [3] and as a preferential secondary site for the metastasis of primary carcinomas such as breast, prostate and lung cancers [4,5]. Paracrine factors are secreted by or released from many components of this microenvironment including the mineralized bone matrix, the major skeletal cells (e.g. osteoblasts and osteoclasts) and the cells of the bone marrow. Through the resorptive activity of osteoclasts, stored growth factors such as transforming growth factor-β (TGF-β) and insulin-like growth factor 1 (IGF-1) can be liberated from the bone matrix [6,7]. Both TGF-β and IGF-1 can act as tumor promoters by enhancing proliferation of malignant cells directly or through the loss of growth inhibition [8-10]. TGF-β is also a profound modulator of the chemical and structural properties of the bone microenvironment where it can: 1) support the degradation of the ECM through activation of matrix metalloproteinases such as MMP-2 [11,12]; 2) induce angiogenesis [13,14] and 3) impair immune surveillance and detection of malignant cells [15,16].

The cells of the bone marrow include hematopoietic stem cells that give rise to blood cell types such as leukocytes and erythrocytes as well as adherent stromal cells such as endothelial cells, fibroblasts, adipocytes and osteogenic precursors. This component of the bone microenvironment is a rich source of chemokines, cytokines and growth and angiogenic factors that support the proliferation and differentiation of these cells [2]. In addition, these factors also promote tumor development in bone. An example relevant to our research is the CCβ chemokine, monocyte chemoattractant protein-1 (MCP-1/CCL2), which was originally characterized for its role as a promoter of monocyte/macrophage migration to inflammatory sites [17] and has been shown to be secreted by endothelial cells, osteoblasts and osteoclasts within the marrow [18-20]. MCP-1 can promote monocyte and macrophage infiltration into various tumors, similar to its function in inflammation, potentially creating a path for further invasion [21]. This chemokine also appears to enhance proliferation of prostate cancer cells and promote the migratory and invasive behavior of prostate as well as breast carcinomas [19,22]. Within this microenvironment, it may participate in a chemokine-driven vicious cycle where MCP-1 can be secreted by tumor cells which subsequently promotes the secretion of TNF-α as well as other promalignancy factors [1]. Since other cellular components such as primary afferent sensory nerves also reside in the bone-tumor microenvironment [23-25], it is interesting to note that MCP-1 can also directly enhance the excitability of such nociceptive afferent neurons [26] and, in this way, might modulate the pain associated with bone metastases.

Deciphering the complexity of interactions between the cells, their paracrine factors and the downstream signaling within the bone-tumor microenvironment that promotes tumor progression and subsequent bone cancer pain continues to be a challenge. It is accepted that a thorough understanding of these interactions is important for the design of future treatment therapies [1]. Therefore, models that can accurately recapitulate various facets of this microenvironment and the multi-cellular interactions present are needed. The current *in vivo *and *in vitro *models have advantages and disadvantages. *In vivo *models provide great insight into tumor progression at the whole animal level but often utilize immunocompromised models and the accessibility of the microenvironment for sampling and analysis of paracrine factors is limited. *In vitro *models usually afford greater ability to manipulate cells and examine molecular and cellular mechanisms but they often lack the three-dimensional architecture and the multiple cell interactions present in the bone-tumor microenvironment. In the studies presented here, we have established an in vitro coculture model to monitor the expression, release and regulation of paracrine factors during the interaction of a bone explant and tumor cells. We show that the bone tissue and a variety of murine and human carcinoma lines are viable in this culture and each secrete factors known to be important in tumor survival. In addition, the interaction of the bone and tumor cells results in distinct regulation of the chemokine, MCP-1/CCL2, and is dependent on an 'intact' microenvironment.

## Methods

### Cell culturing and neonatal femur dissections

#### Cells

Mouse fibrosarcoma NCTC 2472 (ATCC, Manassas, VA) were engineered to express Discoma coral-derived red fluorescent protein (DsRed2) as previously described [27]. The mouse osteosarcoma K7M2 cells were derived from the K12 line through in vivo selection resulting in a highly metastatic line [28] and were provided by Dr. C. Khanna (National Institutes of Health). The parental MDA-MB-231 cells and the bone-seeking MDA-MB-231 BO cells [29] were a gift from Dr. T. Yoneda (University of Texas Health Sciences). All cells were passaged prior to experiments in bone culture media (BCM) which included α-MEM supplemented with 10% FBS, 0.2% BSA, 0.3 mg/ml L-glutamine, 1× NEAA and 1× penicillin-streptomycin and maintained at 37°C in 5% CO_2 _water-jacketed incubator. For experiments, cells were acclimated to low serum conditions by incubation in 5% FBS 24 h prior to plating for cocultures. An additional antibiotic, gentamycin (50 μg/ml), was added to the media when culturing bones or co-culturing bones and tumor cell lines. All media and additional reagents were purchased from Invitrogen (Carlsbad, CA) and Fisher Scientific (Pittsburgh, PA).

#### Neonatal femurs

The outbred, immunocompetent CD-1 strain of mice was used for these experiments (Charles Rivers Laboratories, Wilmington, MA). Mice were bred and femurs from two-day postnatal offspring were collected according to protocols approved by the IACUC committee at the University of Minnesota. Femurs were initially grossly dissected from the limbs and then placed on PBS-soaked paper towels under a dissecting scope. Femurs were carefully stripped of muscles, tendons and loose connective tissue using fine-tipped forceps and microscissors. Small fragments of tendons often remained on the condyles and at the epiphyseal-diaphyseal junction. These fragments were left on the bones since they were difficult to remove without damaging the surface of the diaphysis or tearing off pieces of the condyles (epiphyses). Following dissection, femurs were immediately placed in serum-free BCM with added gentamycin and kept in the incubator prior to coculture setup. Tissues from one neonate were processed in approximately 15 min and dissected femurs were incubated a maximum of 2 h prior to placement in wellplates (as described below), which contained BCM without serum (SF) or with 1% horse serum (1% HS) as appropriate for each experiment. For marrow-depletion experiments, freshly dissected femurs were placed under a dissecting scope, the condyles were punctured with a 27-gauge sterile needle and ~500 μl of sterile Dulbecco's Phosphate Buffered Saline (DPBS) was flushed through the diaphysis. The clearing of the marrow cavity was verified by the color change of the effluent (from pink to clear) and the change in appearance of the diaphysis from dark red to colorless and translucent. Preliminary tests of this methodology were followed by histological analysis of the diaphysis to verify the effectiveness of this procedure in depleting the marrow. Depleted femurs were immediately place in the appropriate wells to initiate the experiment.

### Coculture set-up

**Figure 1:**
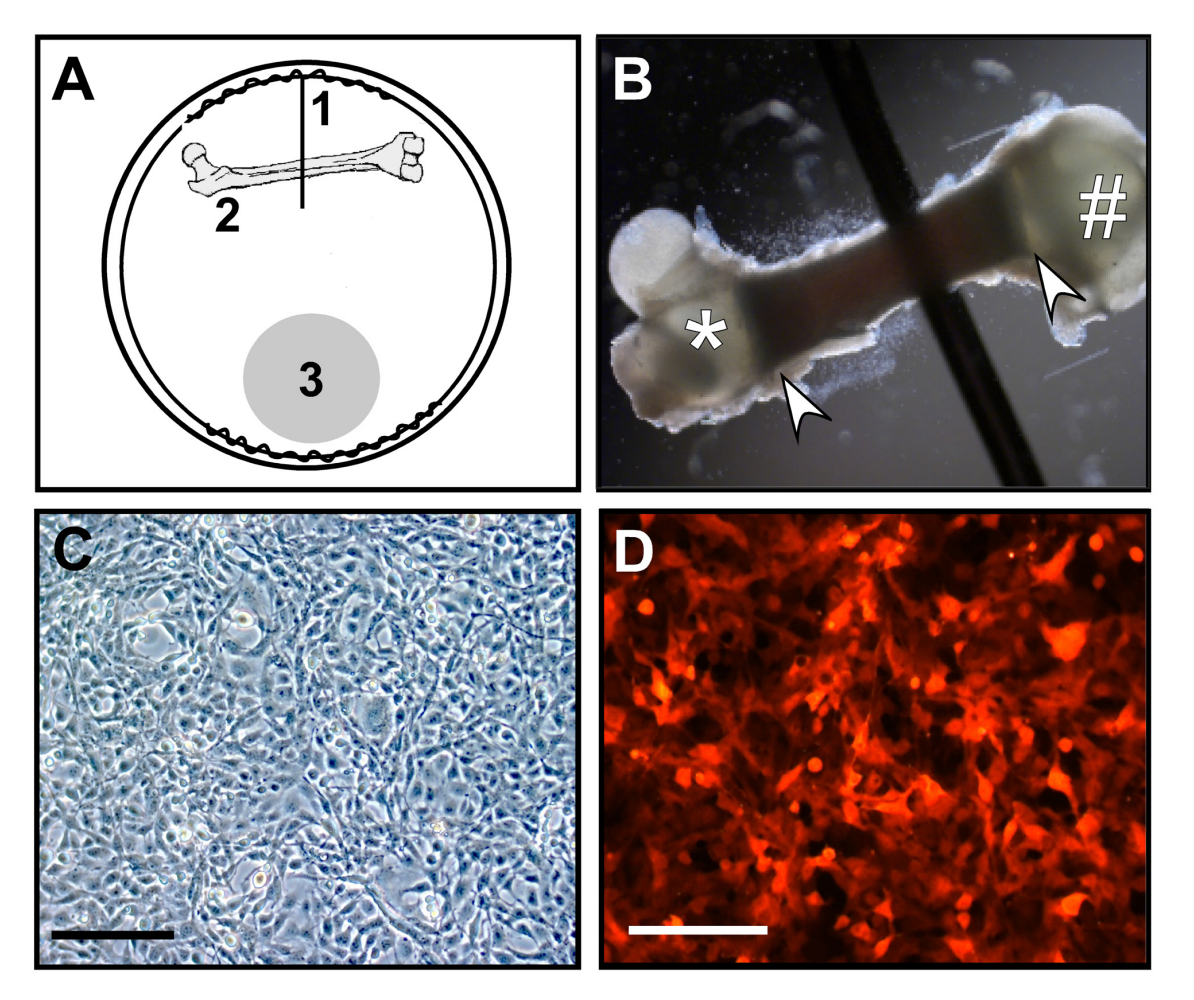
**Characteristics of the bone-tumor cocultures**. **A**. Schematic depicting the placement of the physical and biological components of the coculture system. The stainless steel ring encircles the outer perimeter of each well in a 6-well plate, with a wire protruding from one side into the center (1) that immobilizes a neonatal femur (2). Tumor cells are plated as a bead on the opposite side of the well shown as a shaded circle (3). **B**. Femur is shown in place under the wire with the proximal end (*), the distal end (#) and the diaphysis (arrowheads) visible, as well as the dark marrow cavity. **C & D**. One of the tumor cell lines used in the studies, NCTC 2472 fibrosarcoma cells, are pictured as plated, under brightfield (C) as well as under epifluorescence (D) showing expression of the DSR protein. Bars shown equal 150 μm.

Experiments were conducted in 6-well culture plates where each well was fitted with a braided, stainless-steel ring and wire apparatus as shown in Fig. 1. Each apparatus was made from stainless steel wire wound to form the ring and wire structure, fitting snugly in each well. These forms were cleaned, autoclaved and reused as needed. Incubation of these forms with cells had no effect on the viability of the tumor cells. All tumor cells were plated in a 35 μl bead of ~12,000 cells on one side of each well, within the stainless steel form, and incubated for 2 h to allow for adherence. After this incubation, 3 mls of the appropriate BCM was added to each well followed by the placement of a freshly dissected, single neonatal femur under the wire of the form. Once completed, plates were incubated at 37°C in a 5% CO_2 _water-jacketed incubator and remained undisturbed until the sampling time points indicated for each experiment.

### Viability assays

#### Cell counts

The viability of all tumor cells was assessed at time points indicated by determining total cell number in each well. Cells were trypsinized, transferred to a hemacytometer and cell counts taken, with approximately 4 wells sampled at each time point for calculation of the mean total cell numbers/well and standard error of the means (SEM).

#### Outer bone layers

To determine the percentage of live cells in the outer layers of the dissected bones, an average of 3 femurs were collected at each indicated time point and were labeled using the Live/Dead Viability/Cytotoxicity Kit (Invitrogen/Molecular Probes) with modifications. Femurs were washed in 1 × Dulbecco's phosphate buffered saline (DPBS; w/out Ca^2+ ^or Mg^2+^) and incubated in a DPBS solution containing 8 μM ethidium homodimer-1 and 0.8 μM calcein AM for 1 minute in a microwave processor. Digital images of green fluorescing cells (live cells) and red fluorescing cells (dead cells) were immediately collected on the ventral surface of the sampled femur, encompassing ~1/3 of the bone length including the distal epiphysis and part of the diaphysis with a tissue penetration of ~250 μM. For each femur, a Z-stack of images was captured at 4× magnification using the MRC 1024 confocal microscope system (Biorad, Hercules, CA) and cell counts were determined using Image Pro Plus 4.5.1 (Media Cybernetics, Bethesda, MD). The percentage of live cells was determined by dividing the number of live cells by the total cell number derived from each Z-stack and calculating means and standard error of the means (SEM).

#### Bone marrow cavity

The in situ determination of marrow viability was performed using the TMR Red In Situ Cell Death Detection kit (Roche Applied Science; Indianapolis, IN) on sections of bone tissue. Femurs were collected at each time point, fixed in 10% neutral buffered formalin for 24 h, decalcified in 10 mM EDTA/PBS pH 6.2 for 14 h, paraffin-embedded and sectioned (4 μm). Slides were processed following manufacturer's protocols with positive control slides treated with 50 μl DNase I (3000 U/ml) for 10 min prior to processing. Slides were digitally imaged using a Nikon Eclipse E800M fluorescent microscope. Apoptotic cell counts from the TMR Red stain were taken from four 400 × 400 pixel fields per bone (2 fields/diaphysis; 2 fields/epiphysis) using Image Pro Plus 4.5.1 and averaged across each bone. All means and standard error of the means (SEM) were calculated on an average of 3 femurs per time point.

#### Cytopreparations of bone marrow

Bone marrow was extracted from femurs at each time point as described under the section on neonatal femur preparation above. Briefly, freshly dissected femurs were placed under a dissecting scope in a 60 mm dish and the condyles were punctured with a 27-gauge sterile needle. Sterile DPBS was flushed through the diaphysis until the cavity was cleared, as judged by the color and appearance of the effluent as well as the diaphysis. The extracted marrow was passed through a needle to disperse cells, concentrated by centrifugation (1500 rpm, 5 min) and resuspended in 150 μl DPBS. Concentration onto microscope slides was performed (Shandon Southern Cytospin; 1000 rpm, 5 min) followed by staining using the Hema-3 stain set (Fisher Scientific). Images of slides were captured at 50 & 100× magnification and analysed for cell composition and integrity of marrow.

#### Data analysis

For analysis of viability of tumor cell lines, statistical significance was determined using ANOVA without matching (P value < 0.05) to determine treatment effect and Bonferroni post-tests for selected comparisons between treatments using Prism 4.0 (GraphPad, San Diego, CA).

### Enzyme-linked immunosorbent assays (ELISA)

The concentration of the secreted factors in the BCM was determined using the following Quantikine kits (R&D Systems, Minneapolis, MN): human/mouse/rat/porcine/canine TGF-β1, human/mouse total MMP-2 and mouse JE/MCP-1/CCL-2. Media was collected from an average of 2–3 separate, undisturbed wells at times indicated, centrifuged to eliminate cell debris and stored at -80°C. Samples were assayed in triplicate for these factors following manufacturer's protocols with minor modifications in sample dilutions as necessary. Final protein concentrations were averages of triplicate samples corrected for background media or negative control values. At least three independent experiments were performed and combined means ± standard error of the means (SE) were calculated. Statistical significance was determined using ANOVA without matching (P value < 0.05) to determine treatment effect and Bonferroni post-tests for selected comparisons between treatments.

### Quantitative real-time PCR

To monitor changes in JE/MCP-1/CCL2 expression in the cocultures, quantitative real-time RT-PCR (Q-PCR) was used. For RNA extraction from tumor cells collected in each experiment, 2–3 sets of cells combined from 3–4 duplicate wells were collected at each time point. Cells were trypsinized, cell suspensions combined and centrifuged and pellets stored at -80° for later extraction. For RNA extraction from bones, 2 sets of bones combined from 2 duplicate wells were collected at each time point. Bones were snap frozen in liquid nitrogen and stored at -80°C. RNA extractions were carried out using the RNeasy Kit (Qiagen, Valencia, CA) with minor modifications. To enhance extraction efficiency from bone tissue, individual bones were ground to a fine powder in liquid nitrogen using a mortar and pestle prior to processing. All RNA samples were treated with DNase I using standard protocols (TURBO DNA-*free *kit; Ambion). Complementary DNA was generated from 0.2 μg total RNA using the TaqMan reverse transcriptase reagents (ABI, Foster City, CA). Primers were designed for detection of MCP-1 transcripts and the endogenous control gene, β-actin using Primer 3 software (MIT; frodo.wi.mit.edu) and were tested using RT-PCR to verify the production and size of the amplicons. The primer pairs utilized were: (1) mouse MCP-1: *forward *– 5'-GAAGGAATGGGTCCAGACAT-3', *reverse *– 5'-ACGGGTCAACTTCACATTCA-3'; and (2) mouse β-actin: *forward *– 5'-AGTGTGACGTTGACATCCGTA-3', *reverse *– 5'-GCCAGAGCAGTAATCTCCTTCT-3'. Q-PCR reactions were conducted with SYBR green dye for amplicon detection using manufacturer's protocol for the Brilliant SYBR Green QPCR Master Mix kit (Stratagene, La Jolla, CA). Dissociation curves for each primer set and gel electrophoresis analysis of Q-PCR reactions were used to verify a single amplicon. When possible, multiple lots of cDNA were tested to insure reproducibility. ΔCt scores for MCP-1 transcripts in each sample were normalized using ΔCt scores for β-actin and expressed as the fold change of control vs. treated using the equation, 2^-ΔΔCt^. Statistical analysis was performed using Mann-Whitney non-parametric unpaired t-tests comparing the fold change of each cocultured sample to the fold change value expected if there was no significant change, that is 0.9–1.20. The P-value was set to <0.05. At least two replicates of each experiment were conducted.

## Results

### Establishment of coculture and component viability

The overall goal of this investigation is to develop a simulated bone-tumor 'microenvironment' to examine paracrine factors important in bone cancer progression and pain. The physical set-up of the coculture model was designed within a well of a 6-well plate where each well contains a fitted stainless steel ring which encircles the perimeter of the well and has a wire protruding towards the center of the well (Fig. 1A, label #1). This wire serves to immobilize a femur dissected from a day 2 neonate (Fig. 1A- label #2, B). A femur was chosen as the bone component since the femur is a major site of bone metastases including the sarcoma cells used in the establishment of this model [30-32]. In addition, unlike adult long bone fragments or embryonic calvarial explants often used in other models [33-35], the intact femur retains the three-dimensional, multi-cell architecture including ossified bone and hematopoietic cells, stroma and matrix tissue.

Tumor cells were placed directly opposite of the bone near the edge of the well (Fig. 1A- #3). This location provides separation of the two biological components and prevents contamination of either component during culturing. The NCTC 2472 sarcoma cells were chosen as a fibroblast-like tumor cell that can form painful osteolytic tumors in established murine models of bone metastasis and cancer-induced bone pain [27,30,36,37]. As shown below, we have also successfully cocultured other types of tumor cells. These sarcoma cells and all tumor cells were plated as a bead of media containing ~12,000 cells which results in a highly confluent area of cells as pictured in Fig. 1C. Since the sarcoma subclone used in these experiments expresses the *Discoma *coral-derived red fluorescent protein (DsRed2; Fig. 1D), the location of these cells could be monitored. In addition, since the morphological differences between tumor cells and sloughed cells from the femur is very distinct, we were also able to discern the bone cells from tumor cells. During the culture times examined, spatial separation of tumor cells and bone was maintained.

**Figure 2:**
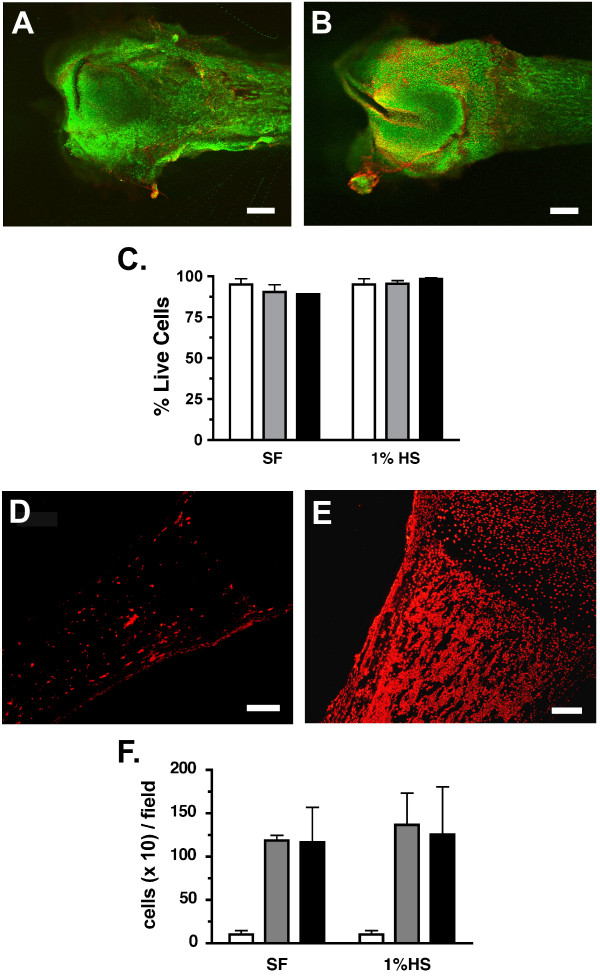
**Viability of femur tissue in culture**. **A & B**. Photomicrograph of femurs freshly dissected (**A**) or cultured in 1% horse serum (HS) for 2 days (**B**). Images are merged z-stacked slices through ~250 μM of the outer surface of the bone. Scale bars equal 0.38 mm (**A & B**). **C**. % live (calcein green) cells in femurs cultured in serum free (SF) or 1% horse serum (1% HS) for 0 days (fresh; white bar), 2 days (gray) and 4 days (black). Data represents an average of 3 femurs per treatment and time point. **D & E**. Photomicrographs of sections taken from freshly dissected femurs assayed for the presence of apoptotic cells (red) within the diaphysis and the marrow cavity (D). Adjacent sections (E) were treated with DNase I prior to TMR staining as described. Scale bars equal 150 μm. **F**. Average number of apoptotic cells per field counted within the diaphysis is shown for fresh BN sections (white bar) and sections from BN cultured for 2 days (gray) or 4 days (black) in SF or 1% HS media.

The viability of each biological component, the femur and the tumor cells, was determined under both serum-free and low-serum (1% HS) culturing conditions. Serum-free culturing eliminated the complication of serum factors and was similar to the normal serum-free conditions in which bone explants are often cultured. Yet, some tumor cells require the presence of serum in vitro for optimal health and proliferation. Therefore, most experiments were performed under both conditions. Bones or sarcoma cells were placed as single components in wells as described above, cultured in serum-free or low-serum media and the viability of each was tested. To evaluate the outer cell layers of the femurs, bones were collected just after dissection (fresh; day 0) and following 2 and 4 days of culture. These explants were then incubated with calcein green (green-live cells) and ethidium homodimer-1 (red-dead cells) and the number of live and dead cells in an ~250 μm optical slice was quantified using confocal microscopy (Fig. 2A, B). The cells labeled within these outer layers would potentially include: loose collagenous tissue, pre-osteoblasts and osteoblasts of the periosteum, chondrocytes within the cartilagenous matrix of the epiphyses and osteoctyes in the developing osteoid of the diaphyses. The percentage of live cells labeled ranged from 89–98% with little change in viability observed through 4 days of culturing, regardless of the media conditions (Fig. 2C). To assess the interior cells of the femur, bones were collected at the same timepoints before and during culturing, processed and stained for the presence of apoptotic cells within the epiphyses (i.e. condyles, ends) and the diaphysis (i.e. shaft) of the bone (Fig. 2D, E). Analysis of sections stained with TMR red labeled nucleotides revealed a significant increase in the numbers of apoptotic cells in the diaphysis and marrow cavity after 2 days of culture in either media condition (Fig. 2F). No change in these numbers was noted at 4 days of culture. In contrast, little to no apoptotic cells (< 5 cells/field) were observed in the epiphyses regardless of condition or time in culture. Bone marrow cytopreparations were also conducted as a qualitative assessment of the viability of the marrow compartment. Prepared smears of fresh, 24 and 48 hr cultured femurs were examined to determine the presence and relative predominance of cell types normally present in this compartment. Marrow isolated from freshly dissected femurs contained a normal representation of hematopoietic lineages, no apoptosis or necrosis, presence of megakaryocytes and the occasional osteoblasts and osteoclasts. Cytopreps from 24-hour cultured femurs revealed increased cell death with the appearance of necrotic and lysed cells, a shift in granulocytes toward segmented neutrophils and the occasional osteoblasts and osteoclasts. After 48 h of culturing, most marrow cells were apoptotic or necrotic with a large reduction in mature granulocyte precursors and segmented neutrophils. The prevalence and viability of osteoblasts and osteoclasts appeared to remain constant through 48 h of culturing. These analyses support the TMR red staining and suggest that the cells of the marrow compartment, in contrast to the outer cell layers of the bone and the epiphyses, are most viable within the first two days of culture. Therefore, to ensure the potential contribution of all cell compartments of the bone tissue, experiments to examine secreted factors in this coculture model were limited to 48 h.

**Figure 3:**
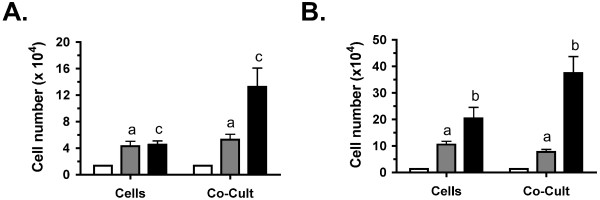
**Viability of fibrosarcoma cells in culture**. Viability of mouse NCTC 2472 fibrosarcoma cells cultured alone (Cells) or with neonatal femurs (Co-Cult) was determined by cell counts. Cell numbers are shown at initial plating (white bars), day 2 of culture (gray) and day 4 of culture (black) in serum-free (SF; graph **A**) and 1% horse serum (HS; graph **B**) media. Across three independent experiments, counts were taken from a total of 5 wells for each time point and media. Day 2 cell counts were found to be significantly different from the initial cell counts for both medias. Lowercase letters indicate comparisons between cells alone and bone-cell cocultures where (**a**) above each bar means no significant difference whereas (**b**) or (**c**) above each bar means a significant difference at P < 0.05 or P < 0.001 respectively.

The viability of the sarcoma cells was determined by evaluating cell numbers. Cells were plated as described and cell counts taken on days 0, 2, and 4 of culture. When compared to initial plating counts, a significant increase in cell numbers was observed at day 2 for cells alone as well as cocultures in both serum-free (Fig. 3A) and low serum media (Fig. 3B). Therefore, these cells are viable and proliferate under these conditions. In contrast, when comparing between cells alone and cocultures at this time point, no significant difference in cell numbers was observed. This similarity in cell numbers makes it possible to compare the differences in secreted factors observed in single component cultures (i.e. cells alone, bone alone) vs. cocultures based on the interactions between components rather than dramatic changes in the number of tumor cells. Additional increases in cell numbers were also observed at day 4 with the trend for higher numbers in cocultures evident for both serum-free and low serum media.

### Regulation of secreted paracrine factors in cocultures

**Figure 4:**
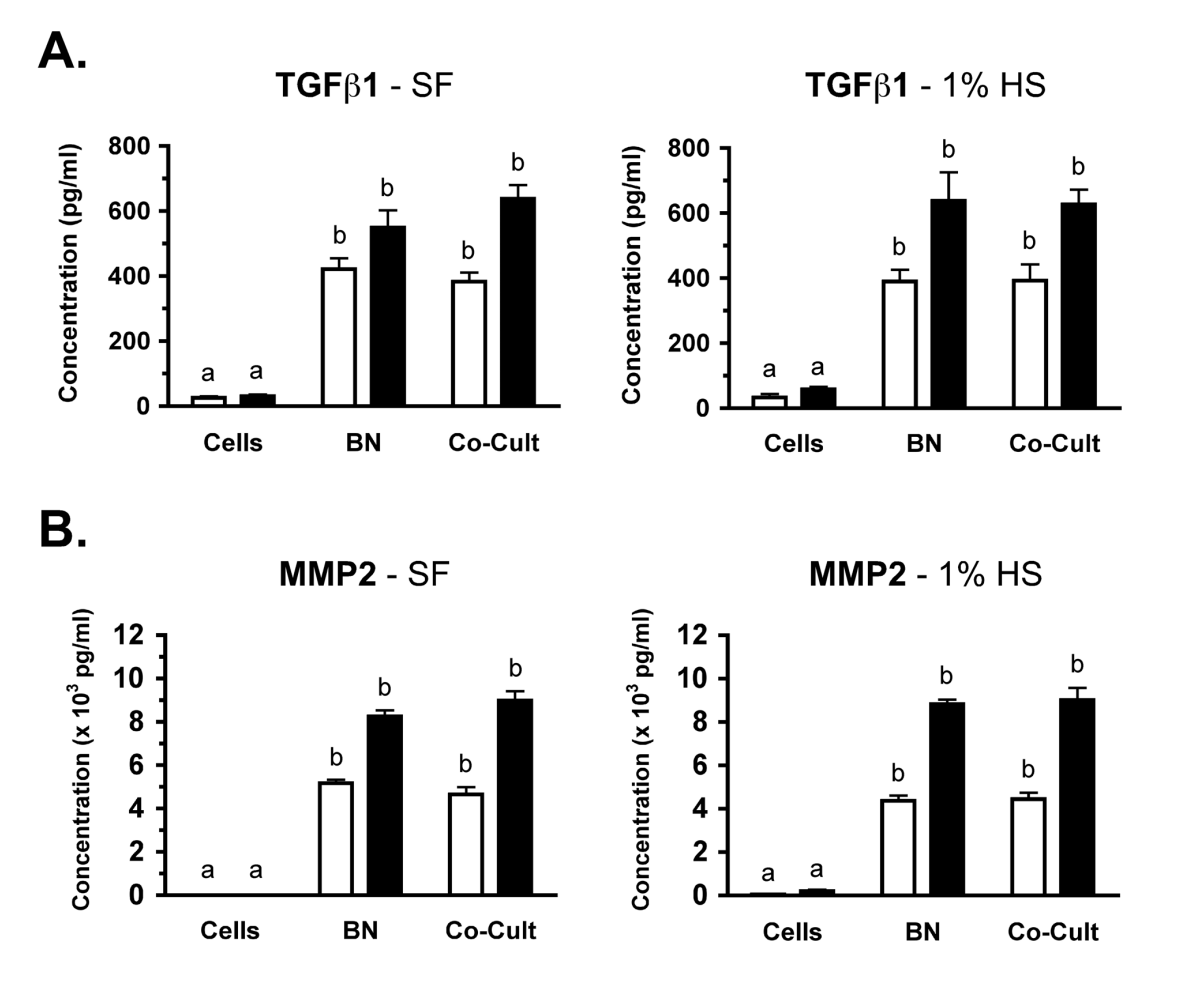
**Secretion of TGFβ1 and MMP2 in cultures**. NCTC 2472 fibrosarcoma cells were cultured alone (Cells), neonatal femurs were cultured alone (BN) or these components were cultured together (Co-Cult) and media was collected at indicated times. Across four independent experiments, media was sampled from 8–10 wells for each time point, culture condition and media. **A: **Media concentration of transforming growth factor β1 (TGFβ1) at day 1 (white bar) and day 2 (black) are shown for both serum-free (left graph) and 1% horse serum (right graph) conditions. Data is graphed as mean pg/ml of TGFβ1 ± SE. **B: **Media concentrations of matrix metalloproteinase 2 (MMP2) at days and media conditions as in A. Data is graphed as mean (× 10^3^) pg/ml of MMP2 ± SE. For A & B, significance of statistical comparisons between single-component cultures and cocultures where (**a**) means no significant difference whereas (**b**) means a significant difference at P < 0.001. Across three independent experiments, media was sampled from 5–6 wells for each time point, culture condition and media.

To characterize the paracrine factors secreted in this model system, the concentrations of transforming growth factor β1 (TGF-β1), matrix metalloproteinase 2 (MMP2) and monocyte chemoattractant protein-1 (MCP-1) were determined in the media of single component cultures and cocultures. Analysis of TGF-β1 revealed that both the cells and the bone secrete or release this protein, with the concentrations observed in bone alone cultures ~16–18 fold greater than cells alone in either media (Fig. 4A; P < 0.001). Therefore, the bone appears to be the major contributor of TGF-β1 to this coculture. In addition, no significant difference was observed in TGF-β1 concentrations between bone alone and bone-sarcoma cocultures. This suggests that there are no interaction effects between the sarcoma cells and the bone regulating TGF-β1 secretion in these cocultures. Analysis of MMP-2 protein revealed that MMP2 concentrations were low (1% HS) to nondetectable (SF) in the cell alone cultures so, as observed for TGFβ1, the bone is also the major contributor of MMP2 to the cocultures (Fig. 4B;P < 0.001). Like TGF-β1, no significant difference between MMP2 concentrations of bone alone and cocultures is observed therefore there appears to be no interaction between the components that influences MMP2 secretion.

**Figure 5:**
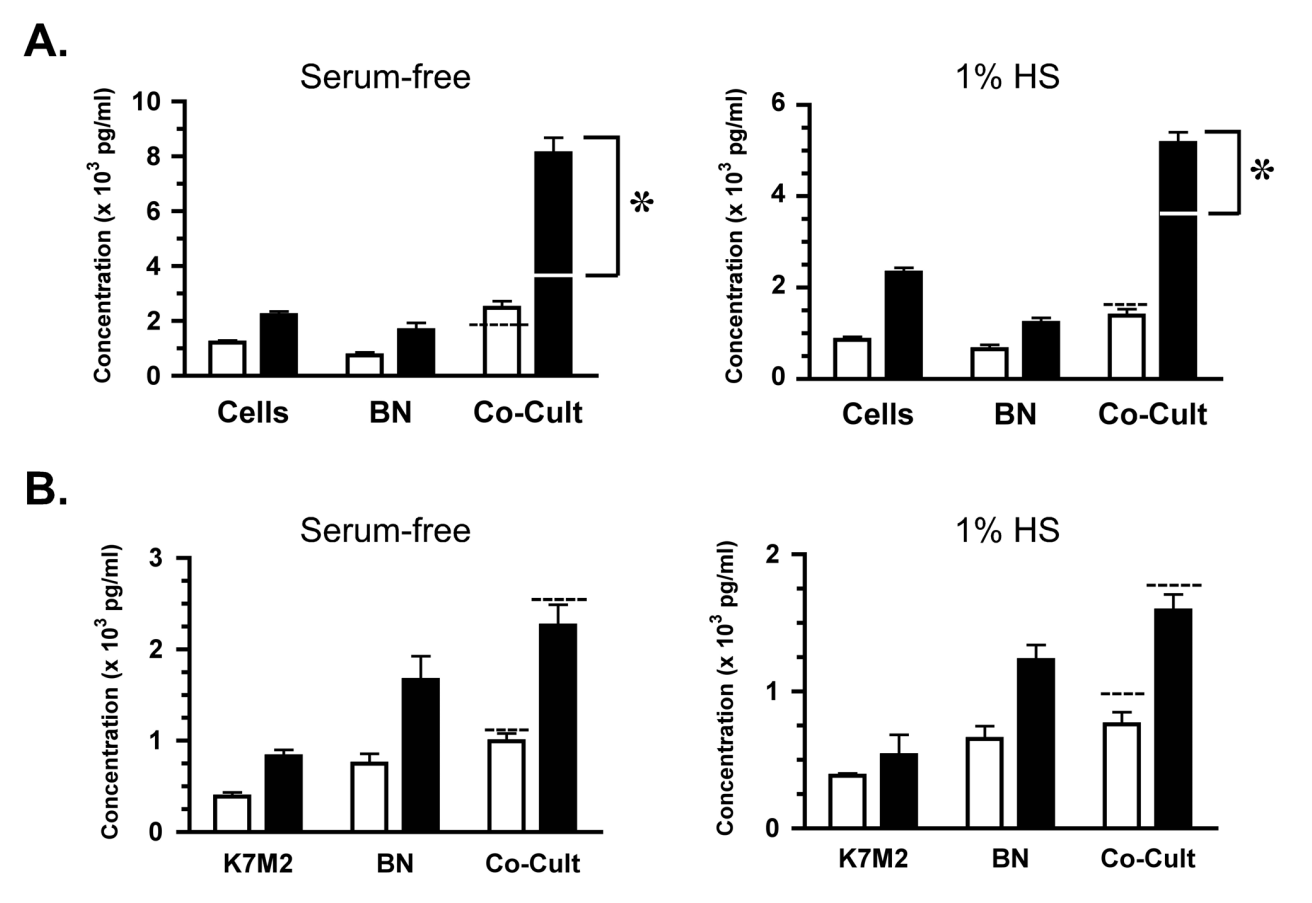
**Synergistic regulation of MCP-1 in cocultures**. NCTC 2472 fibrosarcoma (Cells) or mouse osteosarcoma (K7M2) cells were cultured alone, neonatal femurs were cultured alone (BN) or these components were cultured together (Co-Cult) and media was collected at indicated times. Across four independent experiments, media was sampled from 8–9 wells for each time point, culture condition and media. Mean × 10^3 ^pg/ml of MCP-1 ± SE is shown for day 1 (white bar) and day 2 (black) of culture. **A: **A greater-than-additive increase in MCP-1 concentration in femur-fibrosarcoma cocultures is observed as compared to cultures of femur or cells alone, regardless of media conditions. Simple sum of [MCP-1]cells + [MCP-1]bn is indicated on or above the day 1 Co-Cult bar (dashed line) and on the day 2 Co-Cult bar (white line). Bracket indicates the difference between this sum and the actual [MCP-1] observed in cocultures which is statistically significant at P < 0.001(*). **B: **Cocultures conducted with the mouse osteosarcoma K7M2 cells, known to secrete MCP-1 and interact within the bone microenvironment, showed no synergistic regulation of MCP-1 regardless of culture conditions. Simple sum of [MCP-1] cells + [MCP-1]BN is indicated above the day 1 and day 2 Co-Cult bars (dashed line).

In contrast to these factors, the analysis of MCP-1 concentrations indicated a strong interaction between coculture components. An enhancement of MCP-1 secretion was observed in all femur-sarcoma cocultures (Co-Cult) as compared to each of the single component cultures, regardless of serum conditions or day of culture (Fig. 5A; P < 0.001). Interestingly, the concentration of MCP-1 in these cocultures at day 2 (SF 8115 ± 564; 1% HS 5165 ± 235 pg/ml) was significantly higher than the simple sum of the concentration in the single component cultures (white line on Co-Cult bars in Fig. 5A; BN alone + Cells alone: SF 3875 ± 295; 1% HS 3559 ± 151 pg/ml). This potentiation of MCP-1 secretion suggests a synergistic interaction between the femur and tumor cells in which the release of specific paracrine factors could result in enhanced MCP-1 secretion by one or both of these components. This was observed as early as day 1 in serum-free cocultures (dashed line on Co-Cult bar in Fig. 5A; left graph) and, by day 2, a 1.5–2.1 fold increase in MCP-1 was observed in both media cocultures over the simple sum of sarcoma cells and bone alone (Fig. 5A; P < 0.001). Note that no difference was observed in sarcoma cell numbers in cocultures as compared to single components cultures through day 2 (see Fig. 3A), therefore the strong and significant potentiation observed, especially under SF conditions, cannot be explained by a change in cell number.

To ensure that this observed synergy was not simply a result of the culturing conditions, we cultured another MCP-1 secreting malignant cell line, the K7M2 mouse osteosarcoma cells, with and without femurs. These cells are invasive and highly tumorigenic and can function in the bone microenvironment as both a primary and metastatic tumor [28]. These cells were viable (data not shown) and secrete MCP-1 in both serum-free and 1% HS conditions (Fig. 5B). Like the sarcoma cells, a significantly greater concentration of MCP-1 was observed in cocultures of both serum conditions when compared to single component concentrations (P < 0.01). But, in contrast to the sarcoma cells, no greater-than-additive effect on MCP-1 secretion was observed. The concentration of MCP-1 in the cocultures was not significantly different from the simple sum of MCP-1 secreted by the single component cultures (dashed line above Co-Cult bars in Fig. 5B; P > 0.05). The lack of potentiation of MCP-1 secretion in the K7M2 cocultures suggests that no synergistic interaction occurred between the bone and the osteosarcoma cells. Therefore, it appears that culturing conditions are not necessarily the cause of this synergy. In addition, this regulation of MCP-1 secretion may be unique to the sarcoma cells.

The interaction between the femur and sarcoma cells also resulted in enhanced MCP-1 gene expression. Relative quantitation of MCP-1 transcripts was determined by Q-PCR using cDNA generated from separate RNA isolates of femurs and sarcoma cells cultured alone or cocultured in serum-free conditions. A significant ~1.9 fold increase in MCP-1 transcripts was observed by day 1 in cocultured sarcoma cells as compared to those cultured alone (1.94 ± 0.34 (n = 4); P < 0.05; range = 1.88–2.70). Likewise, in day 2 cocultures, sarcoma cells showed an additional enhancement of MCP-1 expression with an ~3.3 fold increase in MCP-1 transcript levels (3.25 ± 0.24 (n = 4); P < 0.05; range = 2.70–3.48). A modest increase in MCP-1 transcript levels was also observed in cocultured femurs from day 1 cultures (1.31 ± 0.09 (n = 5); P < 0.05; range = 1.11–1.55) but no significant change was observed in day 2 cocultures (0.98 ± 0.08 (n = 5); P > 0.05;range = 0.87–1.15). This data indicates that both the bone and the tumor cells upregulate MCP-1 gene expression with the largest and most consistent upregulation observed in the sarcoma cells. The small increase followed by no change in bone MCP-1 mRNA may indicate that the cells of the bone themselves are not as sensitive to the paracrine effects even though their secretions may modulate the sarcoma cells. As such, this suggests that the sarcoma cells may be the major contributor to the enhanced MCP-1 secretion observed in the cocultures. Also, this expression data provides further support for the conclusion that the bone and tumor cells interact in this model and are able to modulate the secretion and gene expression of paracrine factors from their shared component in coculture.

### Relevance of the bone marrow compartment

**Figure 6:**
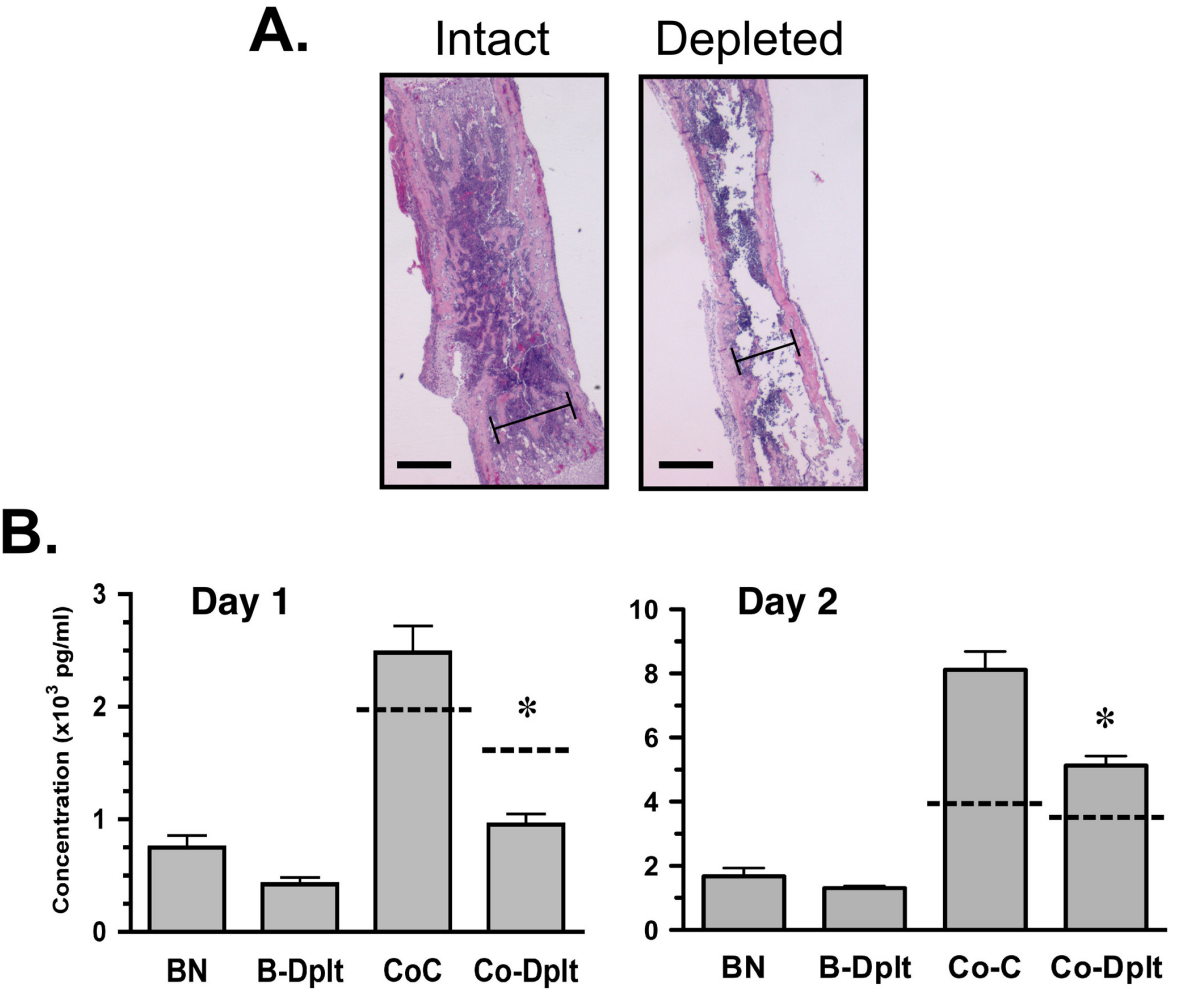
**Marrow depletion attenuates MCP-1 secretion and coculture synergy**. **A: **Photomicrographs of intact and marrow-depleted femurs used in cocultures (4× magnification). The marrow cavity of freshly dissected femurs was flushed using 1 × PBS or left intact and cultured as described. Representative bones were embedded, sectioned and stained with hematoxylin-eosin. Bar = 0.6 mm. Bracket delineates marrow cavity. **B: **Mean × 10^3 ^pg/ml MCP-1 ± SE for day 1 (left graph) and day 2 (right graph) cultures including intact femurs cultured alone (BN), marrow-depleted femurs (B-Dplt), intact femur-fibrosarcoma (Co-C) and depleted femur-fibrosarcoma (Co-Dplt). Dotted line on Co-C and Co-Dplt bar indicate the simple sum of [MCP-1]cells + [MCP-1]BNintact or [MCP-1]cells + [MCP-1]B-dplt respectively. Value for [MCP-1]cells was equal to that shown in Figure 5 as experiments were run in parallel. Across three independent experiments, media was sampled from 7–8 wells for each time point, culture condition and media. Asterisks (*) indicate statistically significant difference between Co-C and Co-Dplt values at P < 0.001.

An important goal of utilizing a femur explant in this model is to attempt to maintain the multicellular and multistructural properties of the bone that are known to be essential for tumor metastasis and growth. The bone marrow compartment is an excellent microenvironment within the bone, whose hematopoietic, immune and mesenchymal cells secrete many chemokines, cytokines and growth factors that attract tumor cells and support their survival. A concern with a whole bone explant is whether such paracrine factors could diffuse out of the tissue and actually affect the function of the tumor cells in coculture. To address this, the marrow cavity of dissected femurs was flushed with PBS to remove the bone marrow cells and these marrow-depleted bones were cultured for 24 h alone or with sarcoma cells. Considering the strong potentiation of MCP-1 secretion observed under serum-free conditions, experiments were conducted in serum-free media. The concentration of MCP-1 in culture media was analyzed and compared to parallel cultures of intact femurs and their cocultures. Histological examination of intact vs. depleted bones revealed removal of a majority of the marrow with little damage to trabecular structure or endosteal surfaces (Fig. 6A). Marrow depletion resulted in a 43% decrease in MCP-1 in bone cultures (BN: 754 ± 107 vs. B-Dplt: 429 ± 60 pg/ml; P < 0.05) as well as a 62% decrease in cocultures (Fig. 6B left graph; CoC: 2487 ± 236 vs. CoC-Dplt: 959 ± 96 pg/ml; P < 0.001). The enhancement of MCP-1 secretion previously observed in the cocultures as compared to intact femurs alone was retained in the depleted femur cocultures (Fig 6B; P < 0.001). However, marrow depletion abolished the potentiation of MCP-1 secretion. In fact, the MCP-1 concentration in cocultures was actually much lower than the simple sum of the depleted femur plus sarcoma cells alone (Fig 6B; dashed line above Co-Dplt bar). This suggests that not only did marrow depletion eliminate potentiation within the first 24 hrs, but it also resulted in reduced MCP-1 secretion from the sarcoma cells. At 48 hours, depleted femurs exhibited a 22% reduction in MCP-1 (BN: 1671 ± 262 vs. B-Dplt: 1308 ± 59 pg/ml; P < 0.05) and cocultures showed a 37% reduction (Fig. 6B right graph; CoC: 8120 ± 564 vs. CoC-Dplt: 5129 ± 294 pg/ml; P < 0.01). These observations indicate that the potentiation of MCP-1 secretion is present in the 48 hr cocultures but is significantly attenuated by marrow depletion. Therefore, regulation of MCP-1 secretion is highly dependent on paracrine factors contributed by the marrow cells early in the interactions between the femur and the tumor cells. In addition, other cell compartments of the femur must contribute during the 48 hr of coculture to maintain the observed MCP-1 secretion.

### Modulation of bone by breast carcinoma cells

**Figure 7:**
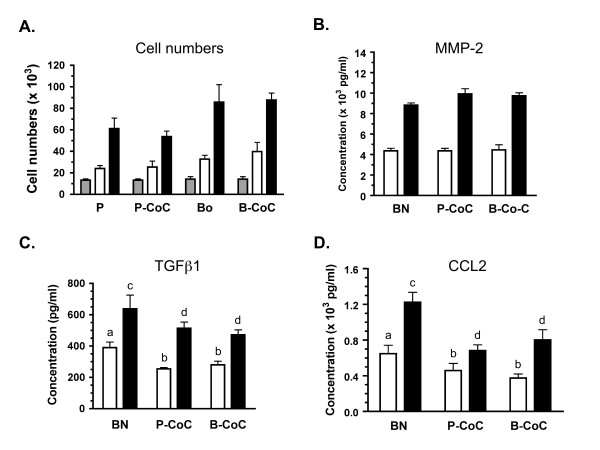
**Cocultures of breast carcinoma cells and mouse femurs show changes in MCP-1 secretions**. The human breast adenocarcinoma parental cell line, MDA-MB-231, and the boneseeking subclone known as MDA-MB-231 BO, were cultured alone and with mouse femurs. Viability of these cells in culture, as assessed by cell numbers, and analysis of secreted factors was conducted as described. Note that no detectable expression of these factors was observed in single cultures of parental or bone subclones. **A: **Cell numbers (× 10^3^) are shown for the parental line (P), the parental-femur coculture (P-CoC), the bone subclone (Bo) and the Bo-femur coculture (B-CoC) at the initial plating (gray bar) and day 1 (white) and day 2 (black) of culture. **B-D: **Concentration of MMP2 (**B**), TGFβ1 (**C**), and MCP-1 (**D**) is shown for the femur alone (BN) and the cocultures at day 1 (white) and day 2 (black) of culture. In C & D, significant differences between concentration of the secreted factors observed in BN cultures (**a **or **c**) compared to cocultures with parental or bone-specific MDA cells is indicated by a different letter above the Co-C bars, with significance at P < 0.01 (**b**) or P < 0.001 (**d**). Across three independent experiments, media was sampled from 7–8 wells for each time point, culture condition and media.

The appropriateness of this model for human carcinoma cells was tested using the parental MDA-MB-231 estrogen receptor negative breast adenocarcinoma cell line [38] and a bone-seeking clonal line produced from these cells known as MDA-MB-231 BO [29]. Both lines showed optimal viability in 1% HS culturing conditions so only this serum condition was used in subsequent experiments. Cell numbers revealed that parental and BO lines proliferated well from initial plating through day 2 and no significant differences were noted between cultures of cells alone and cocultures with femurs (Fig 7A). Analysis of secreted paracrine factors revealed that MMP-2, TGF-β1 and MCP-1 were not detectable in cultures of parental (P) or bone-seeking (BO) cells alone, but were present in femur alone cultures and cocultures in concentrations comparable to those observed in sarcoma cultures (Fig. 7B, C, D; also see Fig. 4, 5). The bone tissue was the major source of these proteins in the breast carcinoma cultures and, in the case of MMP-2, no significant difference between single component cultures (cells or femurs alone) and the cocultures was observed (Fig. 7B). Interestingly, in contrast to MMP-2, secretion of both TGF-β1 and MCP-1 from the femurs was attenuated in the presence of the P or BO cells (Fig. 7C, D). MCP-1 secretion was most dramatically reduced in both P and BO cocultures, with the highest reductions of 46% observed in the day 1 cocultures of the BO line (P < 0.05) and 44% in day 2 cocultures of the P line (Fig. 7D; P < 0.001) as compared to cultures of femurs alone. The secretion of TGF-β1 was also reduced by both tumor lines, with the greatest reductions observed on day 1 of P cocultures (35%) and BO cocultures (28%; Fig 7C; P < 0.05). These results revealed that the MDA-MB-231 breast carcinoma cells can modulate the secretion of paracrine factors from the bone explant.

## Discussion

Modeling the unique characteristics of the bone-tumor microenvironment is an important endeavour for our understanding of tumor progression and metastasis as well as the development of bone cancer pain. We have established a coculture model that simulates many of the facets of this microenvironment and affords several advantages. Those advantages include: (1) the maintenance of the complexity and relevance of the bone component of this 'microenvironment'; (2) the distinct separation of the biological components to allow accurate analyses of each component; and (3) the ease of *in vitro *manipulation of all biological components. The bone component of this model, the intact femur explant, retains the three-dimensional, multi-cellular architecture consisting of ossified bone, hematopoietic cells, osteoblast and osteoclast precursors, stroma and matrix tissue. Our analyses suggest that the femur is viable in these cultures and is capable of active and regulatable secretion of important paracrine factors. The cells of the marrow cavity encased in the diaphysis of this tissue are important contributors to this model since loss of this compartment via marrow depletion attenuates MCP-1 secretion from bone and abolishes the potentiation due to bone-sarcoma interactions. This suggests that paracrine factors are secreted by these marrow cells, interact within this compartment and diffuse into the culture to modulate the tumor cells. Other in vitro models which have been used to test the influence of secreted bone factors on tumor growth and survival or the paracrine effects of tumor cells on bone resorption often employ single cell cultures, calvarial bone or long bone fragments, all of which have little to no marrow component present [33-35]. It is true that the bone marrow contribution to this model would be presumed to be limited to the early interactions of the bone and tumor since its viability declines after 24 h. Even with this limitation, a modulation of a paracrine factor due to component interactions can be observed and that modulation is significantly affected within this time frame by the loss of the bone marrow.

The presence of the femur in cocultures with the sarcoma cells results in a strong up- regulation of MCP-1 expression and secretion. Within 24 h, a greater-than-additive increase in MCP-1 concentrations is observed along with increased MCP-1 mRNA in both femur and sarcoma cells and this potentiation is greater in 48 hr cocultures. These results suggest that paracrine and autocrine regulation of MCP-1 occurred in the cocultures due to the action of factors released by the bone, the tumor cells or both components. Numerous factors, known to be present in the bone-tumor microenvironment, have been shown to induce MCP-1 secretion and gene expression including MCP-1 itself [39], TGF-β [40,41], tumor necrosis factor-alpha (TNF-α) [42], parathyroid hormone related peptide (PTHrP) [39] and various interleukins [43,44]. The mechanism for this potentiation and the factors responsible in our model are currently under investigation.

It is also interesting to note that comparable concentrations of MCP-1 protein secreted by these sarcoma cells in this model have been observed by our research group in *in vivo *mouse models of bone cancer pain. Microperfusates collected in situ from tumors generated by these fibrosarcoma cells contained approximately 4–7 ng/ml [45] which is a range similar to the 5–8 ng/ml observed in the 48 hr bone-tumor cocultures. In addition, strong MCP-1-like immunoreactivity is detected in tumor biopsy samples [45]. These results suggest that our model can accurately simulate this microenvironment, recapitulating the regulation of this chemokine and potentially other paracrine factors. One future direction for our research is to expand this coculture model to include neurons dissociated from dorsal root ganglia (DRG). This will allow the analysis of local chemical mediators contributing to the production of bone cancer pain which is difficult to conduct in *in vivo *mouse models. Research to date has shown that DRG neurons express receptors (CC chemokine receptor 2 and 4) activated by MCP-1 [46], and that lack of CCR2 receptor can impair inflammatory and neuropathic pain responses [47,48]; all supporting a potential role for MCP-1 for the unique type of pain elicited at the site of bone cancers.

In contrast to the upregulation of MCP-1 in the sarcoma cultures, it was also observed that co-culturing with the breast carcinoma cells results in a robust reduction in TGF-β and MCP-1. This suggests that bidirectional communication between the bone and tumor exists in this model where secretions from the tumor cells can regulate the release or secretion of paracrine factors from the bone tissue. The MDA-MB-231 cell lines did not secrete detectable protein in the single-component cultures and human-specific assays did not detect either cytokine in cocultures. So, it is presumed that both are being actively secreted from cells within the bone tissue or passively released by bone resorption during culturing. Transforming growth factor-β is one of the most abundant growth factors stored within the mineralized bone matrix [6,7] and some degradation of the cortical surface of the femur was observed after 48 h of culturing. Passive release of TGF-β is therefore possible in these cocultures and factors released by these breast carcinoma cells may be modulating this process. In contrast, although it can be stored in secretory granules of endothelial cells prior to active secretion [49], MCP-1 is not known to be stored within the bone matrix. Therefore, paracrine factor(s) released by the breast carcinoma cells may regulate the active secretion of MCP-1 from cells of the femur tissue which could include osteoblasts, marrow endothelial cells or immune cells such as monocytes [18-20]. It is interesting that both of these cytokines are reduced in parallel raising the possibility that this effect of the femur-carcinoma interactions could reflect linked regulation. It has been shown that TGF-β can have growth-inhibitory and apoptotic effects on normal and malignant cells, including MDA-MB-231 cells [50,51]. In addition, TGF-β can enhance MCP-1 expression and secretion from a variety of cell types including osteoblasts and immune cells [40,41,52]. Therefore, during the early interactions in the cocultures, the MDA cells may counter any potential growth inhibition by suppressing TGF-β secretion. A decrease in TGF-β in this microenvironment may cause a loss of positive regulation of MCP-1 secretion, due to direct effects of TGF-β or through changes in the secretion of other paracrine factors.

Like all experimental approaches, this simulated model of the bone-tumor microenvironment has certain limitations that need to be considered. First, it is most applicable for the investigation of early paracrine interactions between tumor and bone which are not mediated by cell-cell or cell-matrix interactions. The contribution of the hematopoietic compartment to this model are limited to the first 24–48 hr of culture. Also, the tumor and the bone are isolated from each other. The advantage of this is that it allows the dissection of short-term, acute responses to secreted factors in this microenvironment but does not address the role of such factors with chronic exposure. Second, the neonatal femurs utilized have a greater amount of cartilage than would be expected in an adult femur. This age of femur was chosen to improve the ease and efficiency of dissection as well as optimize tissue permeability. Such a difference in bone explants from adult bone, where human primary and metastatic cancers most often reside, is also the case for research conducted on cocultures of neonatal calvaria and breast or prostate carcinomas [33,34]. Therefore, the assumptions for extrapolation of such results to the disease process would be similar but our model would afford a more complex and representative microenvironment. Finally, as presented here, the major tissue or cell source of paracrine factors modulated by the bone-tumor interactions can only be presumed primarily due to the complexity of the bone tissue. But, in future experiments, this limitation can be eliminated by the use of tumor cell lines or tissues from transgenic animal models where the expression of these factors has been manipulated.

## Conclusion

Our research establishes the usefulness of a coculture model to study the interaction of paracrine factors between tumor cells and the 'host' cells of the bone microenvironment. Studies with this model have revealed its appropriateness in simulating the regulation of the cytokine MCP-1 in interactions between sarcoma cells and bone. These interactions result in the enhancement of MCP-1 secretion which is dependent on the presence and viability of the bone marrow compartment as well as other cells of the bone tissue. Other paracrine factors such as TGF-β and MMP-2 are secreted but are not regulated by these bone-tumor interactions. The observations with breast carcinoma cells in these cocultures supports the applicability of this model for multiple types of tumor cells. The interaction of these carcinomas with femurs resulted in the downregulation of both MCP-1 and TGF-β secretion from bone, revealing distinct patterns of regulation of these factors and 'cross-talk' between these tissues. This coculture system should provide further insight into the paracrine interactions between malignant cells and the bone microenvironment that are involved in the progression of sarcomas, which are poorly understood, as well as the metastasis of other cancers.

## Competing interests

The authors declare that they have no competing interests.

## Authors' contributions

KRS conducted all coculture studies, performed data collection and statistical analyses and drafted the manuscript. MRZ and JA did initial experiments to establish physical setup of cocultures and validated bone viability assays, ELISA and Q-PCR analyses. DLB trained and assisted KRS with cytopreps of bone marrow and evaluated smears for marrow cell viability. AJB assisted in the conception and design of the studies, in the interpretation of the data and in the drafting of the manuscript. LJM was responsible for the conception, design and direction of the studies and actively participated in data interpretation, statistical analyses and drafting of the manuscript. All authors had the opportunity to review and approve the final manuscript.

## Pre-publication history

The pre-publication history for this paper can be accessed here:

http://www.biomedcentral.com/1471-2407/9/45/prepub
